# An Adolescent Female With Acute Abdominal Pain: A Rare Case in Pediatrics

**DOI:** 10.7759/cureus.36296

**Published:** 2023-03-17

**Authors:** Maria Elena Pérez-Arenas, Cristina De Miguel Cáceres, Paloma Ferreiro-Mazón García-Plata, Pedro Luis Pérez-Hernández, Paula García Sánchez

**Affiliations:** 1 Pediatrics and Child Health, Hospital Infantil La Paz, Madrid, ESP; 2 Pediatric Emergency Medicine, Hospital Infantil La Paz, Madrid, ESP; 3 Emergency Department, La Paz University Hospital, Madrid, ESP

**Keywords:** adolescents, sexually transmitted diseases, acute abdomen, pelvic inflammatory disease, abdominal pain

## Abstract

We present the case of a 13-year-old female with a 48-hour history of diffuse abdominal pain, fever, nausea, and vomiting, with worsening in the last few hours. On examination, she had signs of acute abdomen, and laboratory tests showed elevated acute phase reactants (APR). Abdominal ultrasound excluded acute appendicitis. A history of risky sexual behavior was reported, so pelvic inflammatory disease (PID) was considered. Although appendicitis is the most common cause of acute abdomen in adolescents, PID should be suspected in adolescents with risk factors. Prompt treatment is necessary to avoid possible complications and sequelae.

## Introduction

Abdominal pain is a frequent reason for consultation in the pediatric emergency department. Symptoms are usually self-limiting, secondary to minor pathologies (gastroenteritis, constipation, etc.), and only a small percentage requires hospital admission or surgery [1]. However, in adolescent females, certain pathologies should be considered and require a particular diagnostic and therapeutic approach.

Pelvic inflammatory disease (PID) is a polymicrobial infection, which affects the uterus, ovaries, and other female reproductive organs. In most cases, it is considered a sexually transmitted disease, and* Neisseria gonorrhoeae*,* Chlamydia trachomatis*, and *Mycoplasma genitalium* are the commonly involved microorganisms. The main risk factors for its development are the period of adolescence, sexual promiscuity, immunological immaturity, or risky sexual practices [2,3].

The latest data from Western countries show that about 1%-2% of sexually active females aged 15-24 years are diagnosed with PID, and this incidence is higher in the USA and in lower socio-economic classes [2].

Clinical presentation varies widely, ranging from asymptomatic cases to severe symptoms with intense abdominal pain, hemodynamic instability, and shock. The most frequent symptoms are hypogastric pain, dysuria, metrorrhagia, and leukorrhea. Fever and gastrointestinal symptoms (nausea, vomiting, or diarrhea) may be present. The Centers for Disease Control and Prevention (CDC) established diagnostic criteria for PID in 2015, although these are not always present and the diagnosis may not be simple [4]. The most specific diagnostic criteria are laparoscopic findings, though laparoscopy is not indicated except for doubtful severe cases or torpid evolution [5]. It is therefore important to establish a diagnosis of suspected PID in sexually active females with risk factors in order to initiate a prompt treatment and avoid possible complications or sequelae such as chronic pelvic pain, ectopic pregnancy, or infertility.

## Case presentation

In June 2022, a 13-year-old female patient presented to the pediatric emergency department of a tertiary hospital with a fever of up to 38.9°C, diffuse abdominal pain, diarrhea, and vomiting for two days, with worsening in the last few hours. She had no dysuria, but a foul-smelling leukorrhea during the last two weeks was reported.

On admission, she was febrile, her blood pressure was at the low limit of normality, and the rest of her vital signs were normal. Physical examination revealed a decreased level of consciousness, with somnolence and difficulty to focus her attention. She appeared pale and referred to diffuse abdominal pain pronounced over the hypogastrium and right iliac fossa. McBurney's sign was positive. On re-interrogation, she reported heavy consumption of distilled alcohol and drugs (cannabis and ecstasy) during the last 48 hours.

Her last menstrual period occurred two weeks prior to consultation. The patient confirmed unprotected sexual intercourse with five different partners in the last month. On the other hand, the family reports disruptive behavior in recent months, with runaways from home, school abstinence, thefts, etc., with difficult handling and family disruption.

Blood tests showed an elevated leukocyte count (20,880/mcL) with neutrophilia, elevated C-reactive protein (374.9 mg/L), altered coagulation (prothrombin activity of 33%; international normalized ratio {INR}: 3), and normal serum levels of transaminases. Intravenous fluids were started, and a dose of vitamin K was administered. An abdominal and pelvic ultrasound was performed with no data suggestive of appendicitis but with free fluid in the Douglas space. Due to the previous history, microbiological study was completed to rule out sexually transmitted infections (genomic amplification in urine and serological blood study), and a sample of endocervical exudate was collected. This technique showed increased endocervical tenderness. Her urine beta-human chorionic gonadotropin (βhCG) was negative, urinalysis and stool studies showed no evidence of infection, and the urine toxicology screen was positive for tetrahydrocannabinol (THC). Considering the laboratory alterations and the patient's condition, she was started on intravenous antibiotics (ceftriaxone and metronidazole) and oral doxycycline, and she was admitted to the hospital.

Subsequent studies revealed the isolation of *N. gonorrhoeae* in endocervical exudate and positive genomic amplification in urine for *C. trachomatis*,* N. gonorrhoeae*,* Mycoplasma hominis*, and *Ureaplasma parvum*. Serological tests for HIV, herpes, hepatitis, and syphilis resulted negative. During her admission, the clinical and analytical evolution was favorable, with the normalization of analytical parameters. After four days of hospitalization, voluntary discharge was requested due to social problems, and oral antibiotic therapy with doxycycline and metronidazole was indicated for two weeks, with subsequent outpatient follow-up in consultations.

## Discussion

Acute abdominal pain in adolescent females constitutes a diagnostic challenge when differentiating between gastrointestinal causes and gynecological pathology. Our case exemplifies an uncommon cause of abdominal pain in pediatrics, with potential sequelae if prompt diagnosis and treatment are not carried out.

Acute appendicitis is the pathology that may raise the most diagnostic doubts. Its annual incidence is about 100 per 100,000 people, being more frequent between 10 and 19 years of age [3]. It typically presents with diffuse periumbilical pain that later focuses on the right iliac fossa (deep tenderness at McBurney's point), accompanied by fever, nausea, and vomiting. On examination, palpation in the left iliac fossa may induce referred pain on the right side (Rovsing's sign), in some cases suggesting bilateral pain and adnexal pathology. In both situations, it is typical to find leukocytosis with neutrophilia and elevated acute phase reactants (APR) in laboratory tests.

In these cases, a detailed anamnesis is essential. Some factors, such as risky sexual behavior, the presence of leukorrhea, pain on cervical manipulation, or abnormalities in the urine analysis, should make us rule out gynecological pathology.

Imaging tests play an important role in the differential diagnosis. Ultrasound is usually the first test to be performed, and computed tomography (CT) is the one that shows the most defined images. To add further complication to the matter, periappendicitis may develop in the context of salpingitis or PID, with inflammation of the appendiceal serosa without mucosal involvement. Furthermore, a perforated appendicitis may cause leukorrhea in adolescents.

When PID is suspected, prompt diagnosis and treatment are essential to prevent the development of complications and sequelae, such as infertility, ectopic pregnancy, chronic pelvic pain, recurrent infections, sepsis, intestinal obstruction, and increased risk of some ovarian tumors. The latest studies show that *C. trachomatis* is the cause of more serious conditions, with a higher percentage of hospitalizations and sequelae, and early prevention and treatment are imperative [6,7].

Finally, it is important to consider the social problems that may accompany this type of pathology in adolescent females. For this reason, close follow-up and a multidisciplinary approach are essential, requiring the intervention of pediatricians, gynecologists, social workers, and even psychologists.

## Conclusions

Acute abdominal pain in adolescent females is a diagnostic challenge, and it is necessary to rule out gynecological pathology in certain situations. Our case exemplifies a form of PID with significant clinical involvement with abdominal pain and marked elevation of infectious analytical parameters, probably due to the delay in consultation. The presence of these data, together with a history of risky sexual behavior, led to a suspected diagnosis. The evolution after starting antibiotics was favorable. It is essential to suspect this pathology, whose incidence is increasing, in sexually active adolescent females with abdominal pain, as early diagnosis and treatment are essential to avoid complications and long-term sequelae. Prevention in adolescents is fundamental, and a multidisciplinary approach is necessary.
